# How Group Perception Affects What People Share and How People Feel: The Role of Entitativity and Epistemic Trust in the “Saying-Is-Believing” Effect

**DOI:** 10.3389/fpsyg.2021.728864

**Published:** 2021-09-22

**Authors:** Tingchang Liang, Zhao Lin, Toshihiko Souma

**Affiliations:** ^1^Graduate School of Social Sciences, Hiroshima University, Hiroshima, Japan; ^2^Junior College, Fukuoka Institute of Technology, Fukuoka, Japan

**Keywords:** group perception, entitativity, saying-is-believing, audience tuning, epistemic trust, shared reality

## Abstract

This research investigated how interpersonal communication with a large audience can influence communicators’ attitudes. Research on the saying-is-believing effect has shown that when an individual’s attitude is perceived in advance by a communicator, the communicator tunes the message to the person, which biases the communicator’s attitude toward the person’s attitude. In this study, we examined the conditions under which audience tuning and attitude bias can occur with audiences containing more than one individual. We manipulated communicators’ perceived group entity for a large audience and the audience’s prior attitudinal valence and measured the audience’s epistemic trust. The results showed that communicators tuned their messages to the audience’s attitude when they perceived group entitativity and epistemic trust. Furthermore, tuning the message to the audience was found to bias communicators’ subsequent impressions of the topic in a direction closer to the audience’s attitude. These results suggest that perceiving a large audience as a group influences the subsequent impressions of electronic word-of-mouth product or service communicators.

## Introduction

People now share their experiences with others through social networking services by posting on Internet forums, websites, or blogs. For example, people frequently use microblogging platforms (e.g., Twitter) to share their everyday happenings with networked others (e.g., followers). People also use electronic word-of-mouth (eWOM) in the form of online shopping website reviews (e.g., Amazon) to share their experiences with a product or service with unknown others (e.g., Amazon users).

Although such interpersonal communication requires at least two parties, a communicator and an audience, most previous research has focused on the influence that posting and sharing has on the audience, such as information spreading and decision-making. For example, rapidly spreading information has influenced world events, including the Arab Spring (Russell, 2011) and the 2016 U.S. presidential election (Enli, 2017). An influence on decision-making has also been reported, such as the effect of consumer reviews on comparable book sales (Chevalier and Mayzlin, 2006) and early adoption of new products (Iyengar et al., 2011). However, little attention has been paid to how communicators’ shared experiences influence their attitudes.

Studies of interpersonal communication have shown that tailoring and sharing messages with a particular audience affects the communicator’s subsequent memory and impression of the message topic; this phenomenon is known as the “saying-is-believing effect” (SIB; Higgins and Rholes, 1978). The SIB effect suggests that when a communicator tailors a message to a target audience, sharing the tailored message with the target audience changes the communicator’s subsequent memory and impression. In initial SIB paradigm experiments, participants first read stimulus information describing a target person’s evaluatively ambiguous behaviors (e.g., a behavior that can be perceived as either persistent or stubborn). When participants had been led to believe that the audience liked the target person, they tended to create positive messages about that person to help their audience to identify that person among four possible persons. On the other hand, when participants had been informed that the audience disliked the target person, they were more likely to create negative representations of that person for their audience. This audience tuning (Higgins, 1992) also drove positive or negative distortions in participants’ recall and impression of the original stimulus information, wherein prior impressions or memories of the target person were transformed into more evaluative extremes in the positive (or negative) direction after a positive (or negative) message was created. This finding has been replicated many times within similar interpersonal communication contexts (Higgins and Rholes, 1978; Higgins and McCann, 1984; Sedikides, 1990; McCann et al., 1991; Echterhoff et al., 2005, 2008, 2017; Hausmann et al., 2008; Kopietz et al., 2010). Furthermore, the process by which the SIB effect occurs has been explained using the concept of shared reality (Echterhoff et al., 2009). The successful creation of a shared reality with an audience serves to establish the reliability and validity of a topic, and therefore could drive the audience-tuned bias in communicators’ subsequent memory and impression of the message topic (Echterhoff et al., 2005).

These findings were also replicated while extending the SIB effect to a large audience rather than a single audience. Hausmann et al. (2008) found that in situations with a group (e.g., three-person audience) rather than an individual (e.g., one-person audience) as the audience, the SIB effect occurrence depends on whether communicators were given feedback that the audience correctly understood their message. This supports the conceptual explanation of the SIB effect through shared reality (Echterhoff et al., 2005, 2009). However, even though audience tuning was observed in both Hausmann et al. (2008) one-person audience and three-person audience conditions, whether successful shared reality expectations influence audience tuning or not was left undiscussed. The typical communication task in the SIB paradigm, which was asking communicators to help their audience to identify the target among a set of four possible targets, may have allowed all communicators to identify the audience’s need for the information they included in their message, whether the audience was one person or a group. Thus, the communicators can be assumed to have a high expectation of establishing a shared reality with the audience, which could encourage them to tune messages to a larger audience.

Barasch and Berger (2014) findings address this possibility. They examined how audience size could affect communicators’ willingness to share sales information and found that communicating with just a single audience member encourages people to share information about an upcoming business suit sale; however, communicating with a large audience suppresses communicators’ willingness to share such information. Compared to sharing useful information with a large audience, sharing useful information with a single audience member allows communicators to easily infer the information usefulness to their audience (e.g., “The upcoming business suit sale should be useful for my audience”), which in turn encourages communicators’ willingness to share useful content. We argue that this low cognitive load in inferring the usefulness of information is related to the communicators’ expectation of successfully building a shared reality with their audience. For example, when communicators can easily infer the information usefulness for their audience (e.g., the communicators believe that the upcoming business suit sale would be useful for their audience), they can also identify a specific audience viewpoint (e.g., the audience will think that the business suit sale is useful to them), which should lead to clear expectations for successfully establishing shared reality. Furthermore, communicators with large audiences are more likely to acknowledge multiple viewpoints among audience members (Schlosser, 2005). Thus, compared to a single member audience, the difficulty of identifying a specific viewpoint in a large audience may decrease the expectation of successfully establishing shared reality, which suppresses communicators’ willingness to share useful content. If so, communicators’ information tuning may also be affected depending on the difficulty of identifying a specific viewpoint in a large audience. This study proposed that perception of a consensual viewpoint in a large audience and trust in their understanding of the message should be related to an expectation of successfully establishing a shared reality, which could affect audience tuning. In the following discussion, we argue that whether or not a communicator tunes a message for a large audience depends on the communicator’s perception of entitativity or epistemic trust as a group.

In what cases do communicators perceive that a large audience has a consensus perspective? We believe that the degree to which communicators perceive multiple people as an entitative group affects audience tuning. One group may comprise a collection of individuals, but another group may comprise a collection of people with similar or common viewpoints. A crucial factor in identifying between-group differences is the degree of entitativity (Lickel et al., 2000), which indicates the extent to which a group is perceived as an entity (e.g., a single, unified agent) compared to a mere collection of individuals (Campbell, 1958; Hamilton and Sherman, 1996). Members in a high entitativity group are typically perceived as more similar to each other than those in a low entitativity group (Hamilton and Sherman, 1996). Previous research showed that the process of forming cognitive representations and impressions of a group is influenced by the perceived entitativity of that group. People process information about high entitativity group members as easily as they process information about a single individual (McConnell et al., 1997; Yzerbyt et al., 1998). Thus, when a group is perceived to be high in entitativity, people believe that all group members hold a consensual viewpoint about a specific topic. Conversely, when low entitativity is perceived, people believe that group members have differing viewpoints on a topic.

Given that high entitativity perception leads people to believe that members in a group hold a consensual viewpoint about a specific topic, we expect high entitativity to decrease the difficulty of perceiving a common viewpoint within a large audience; conversely, we expect low entitativity to increase the difficulty. Studies employing the SIB paradigm showed that audience tuning requires communicators to acknowledge the audience’s specific viewpoint (e.g., as in Higgins and Rholes, 1978, where participants were asked to tune their messages to their audience who liked or disliked the target person). These factors influence the likelihood of SIB effects.

As described earlier, whether communicators are likely to tune their messages to a large audience depends on the expectation of successfully establishing a shared reality with the audience. We assume that such expectations could be affected not only by the extent to which communicators perceive the audience as an entitative group but also by how communicators perceive epistemic trust in the audience. Epistemic trust, which has been used as an interpersonal measure of shared reality, is the extent to which communicators feel that they can rely on the audience’s view to create their own judgment (Echterhoff et al., 2005). Echterhoff et al. (2005) found that the audience tuning effect on communicators’ subsequent memory and impression, the SIB effect, depends on the communicators’ sense of epistemic trust in the audience. In such cases, communicators were likely to believe that their messages were understood and accepted by the audience. Follow-up studies provided further evidence supporting the role of epistemic trust, which has been used to assess the experience of shared reality (e.g., Echterhoff et al., 2008; Hausmann et al., 2008; Kopietz et al., 2010). The role of epistemic trust was also replicated in the SIB study group contexts. When communicators reported sufficiently high epistemic trust in the group audience, they exhibited an audience tuning effect on memory and impression of the topic (Higgins et al., 2007).

When communicators do not have epistemic trust in the audience, it is unlikely that a shared reality can be established, so communicators will not actively engage in audience tuning, even when informed of the audience’s prior attitudes. Previous studies found that epistemic trust in the audience is positively related to audience tuning (Schmalbach et al., 2019). When communicators can trust the audience’s epistemic competence, they will tune the message along with the knowledge of the audience to establish a shared reality. Studies have demonstrated that the more communicators perceive that the audience can understand the content, the more specifically they will tune the message content. Rubini and Sigall (2002) found that communicators were more likely to share more details about a political view congruent with the audience’s view, which encourages a high expectation of successfully establishing shared reality, which in turn corresponds with the expectation of being understood by the audience. These findings underline the possibility that such content modification (e.g., sharing more details about the topic) is affected by epistemic trust. If so, this would also support our assumption that audience tuning occurs when communicators expect that they can establish a shared reality with their audience.

We have already predicted that audience tuning is likely when communicators perceive high entitativity in a large audience. However, if the audience is perceived as epistemically untrustworthy, despite their high entitativity, communicators will not expect to establish a shared reality with them. Conversely, if communicators can epistemically trust a large audience perceived as entitative, they will have a greater expectation of establishing a shared reality with the audience.

Based on the above, we assumed that communicators’ perceptions of a consensual viewpoint in a large audience and an expectation that their messages can be understood and accepted by the audience should be related to the expectation of successfully establishing shared reality. When communicators perceive high entitativity and high epistemic trust in a large audience, they would tune their messages to the audience. Conversely, when communicators perceive the audience to be a low entitativity group, or when they perceive low epistemic trust in the audience, audience tuning would be suppressed. Therefore, we proposed that the perception of entitativity and epistemic trust may affect audience tuning and hypothesized that audience tuning would occur only when communicators perceive high entitativity and high epistemic trust in a large audience. If entitativity and epistemic trust affect audience tuning, they should also affect communicators’ subsequent memory and impression of the topic. Thus, we also hypothesized that the SIB effect would occur only when communicators perceived high entitativity and high epistemic trust in a large audience.

This study makes two primary contributions to the literature. First, there are potential benefits of introducing the entitativity concept in studying the SIB effect. Previous SIB studies that focused on a large audience may have led communicators to perceive the audience as an entitative group. For example, in Hausmann et al. (2008) study 1, audience members described as independent (i.e., working alone during the experiment) may have led the participants to perceive low entitativity in their audience group, which in turn could have suppressed the audience-tuned bias. However, in their study 2, when audience members were described as interdependent (i.e., discussing the target together and making a joint decision) and participants were informed through feedback that all audience members correctly understood their messages, the participants may have perceived their audience group to be entitative, and therefore showed audience-tuned bias.

Second, whereas previous studies examined the effects of establishing shared reality on SIB, this study focused on whether expectations of establishing shared reality affected audience tuning. We focused on the SIB effect in the context of communication with a large audience with preliminary audience tuning. We proposed that expectations of establishing shared reality moderate audience tuning, and such expectations are induced by perceiving entitativity and epistemic trust in the audience. In examining these propositions, we argued that, in communication contexts with a large audience, the expectation of establishing shared reality may also influence SIB effect occurrence.

This study aimed to examine whether the effects of tailoring and sharing messages with a large audience on communicators’ attitudinal changes would be moderated by communicators’ perceptions of group entitativity and epistemic trust in a large audience. Consistent with this aim, we propose the following hypotheses.

Hypothesis 1: Only when communicators perceive high entitativity and high epistemic trust in a large audience, would they tune their messages to the audience’s attitude.
Hypothesis 2: Only when communicators perceive high entitativity and high epistemic trust in a large audience, would their memories and impressions of the target be biased toward the audience’s attitude.

To test these hypotheses, we conducted an experiment patterned on the specific SIB paradigm of Hausmann et al. (2008), because both studies share the same focus on group-targeted communication.

## Materials and Methods

### Participants and Design

We estimated the required sample size in advance using the following procedure. We planned to examine the interaction effect between audience attitude, group entitativity, and epistemic trust using a hierarchical multiple regression analysis for audience tuning and SIB. Specifically, we intended to examine the increment in R^2^ due to the addition of the three-way interaction term. Using G^∗^power (Erdfelder et al., 1996) with a type 1 error (α) of 0.05, a power (1-β) of 0.8, and an effect size of 0.15, we estimated the required sample size to be 55. Although previous studies (e.g., Hausmann et al., 2008) have found large effect sizes in audience tuning and SIB, they did not examine those effects moderated by the characteristics of an audience group. Therefore, in this study, we adopted a medium effect size in line with Cohen (1988).

Participants were 57 undergraduate students at a Japanese university (29 women and 28 men), with a mean age of 22.19 years (*SD* = 6.20). All participated in this experiment individually and were compensated with Yen 1,000 (approx. $10 U.S. at the time), and course credit. Participants were randomly assigned to a condition in a 2 (audience attitude: positive vs. negative) × 2 (audience group entitativity: high vs. low) between-participants model. The number of participants in the positive attitude × high entitativity condition and the other three conditions was 15 and 14, respectively. The primary dependent variables were the valence of the message and impression. One participant who exhibited strong suspicion and five participants who could not imagine the experimental scenario were excluded from the analyses.

### Procedure and Materials

**FIGURE 1:**
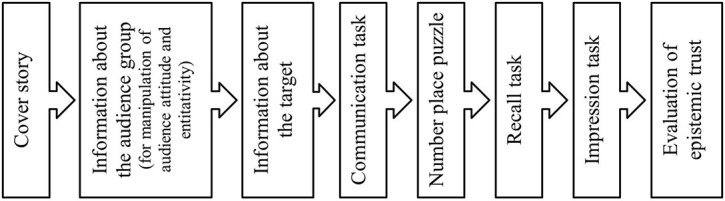
The flow of the experimental procedure.

As this study aimed to examine the SIB effect in the context of communication with a large audience, we patterned our experiment on the specific SIB paradigm that focuses on group-targeted communication (Hausmann et al., 2008). As shown in Figure 1, we first introduced the cover story of the experiment, which required the participants to complete a communication task by creating a message. Then, we provided them with information about the audience group by presenting an evaluation sheet for audience group attitude manipulation, and a discussion memo for audience group entitativity manipulation. In this procedure, participants were randomly assigned to one of the four conditions (audience attitude: positive vs. negative × audience group entitativity: high vs. low). Next, we provided them with information about the communication target using a reference sheet consisting of evaluatively ambiguous descriptions. After all the necessary information had been provided, we instructed the participants to proceed with the communication task. We also asked participants to complete a number place puzzle after the communication task over concerns regarding the effect of short-term memory on the follow-up measures. Then, we instructed the participants to complete the recall and impression tasks, and measured their evaluation of epistemic trust in the audience group’s attitude toward the target.

#### Cover Story

Participants were initially introduced to a communication task (involving a communicator, a target, and an audience group), and told to adopt the role of the communicator. Next, participants were shown a photograph of six undergraduate students (the audience group) who were supposedly participating in part of the experiment. The participants’ task was to describe the target by sending an email to the “audience group” who had already evaluated the target. Based on their descriptions, the “audience group” was to identify the target among a set of four possible targets.

#### Materials

The target information comprised evaluatively ambiguous descriptions, which were used to describe target individuals in previous SIB studies (Higgins and Rholes, 1978; Hausmann et al., 2008), but were modified for this study so that the details described a product (a target laptop) rather than a person. Four descriptions were used in the current study (see Supplementary Figure 1) in response to the characteristics used in previous studies (Echterhoff et al., 2005; Hausmann et al., 2008). We changed the communication topic from a target person to a target product because an eWOM topic (about a product) often involves a large audience. Although most studies on SIB have adopted Higgins and Rholes (1978) initial SIB paradigm—which used evaluatively ambiguous descriptions of a target person (e.g., descriptions of behavior that can be perceived as either persistent or stubborn)—some studies have reported that other stimuli (e.g., products) can also be used as the topic. In Kardes (1986) experiment, bias in subsequent judgments of a product was also found in participants who had read evaluatively ambiguous descriptions of a target product (e.g., descriptions of a stereo that is described to have high performance but low dependability). Therefore, if ambiguity can be ensured in the communication topic (e.g., a topic about an unfamiliar product), the SIB paradigm can be valid for the modified communication topic as well.

#### Audience Group Attitude Manipulation

Before presenting the target laptop information to participants, the experimenter introduced the audience attitude manipulation by presenting an evaluation sheet, whose content was dictated by the audience condition. Participants in the positive audience attitude condition were presented with an evaluation sheet indicating that the audience group had positively evaluated the target laptop (e.g., good in design) (see Supplementary Figure 2); participants in the negative audience attitude condition were presented with an evaluation sheet indicating that the audience group had negatively evaluated the target laptop (e.g., too expensive) (see Supplementary Figure 3).

#### Audience Group Entitativity Manipulation

The audience group entitativity manipulation was then introduced by presenting a discussion memo that recorded the details of the audience group discussing the laptops. The manipulation was designed to comprise several key properties of multidimensional entitativity constructs, including the importance of group membership, interaction among group members, coherence, common goals among group members, and the importance of group membership according to previous research (Newheiser et al., 2012). In the high entitativity condition, the discussion memo indicated that audience group members had an active discussion. Also, the discussion memo stated that they seemed to be coming from the same place, with similar hobbies and common goals according to their conversations (see Supplementary Figure 4). The discussion memo in the low entitativity condition indicated the opposite of the high entitativity condition; they seemed to come from different places and had different hobbies and goals according to their conversations (see Supplementary Figure 5).

#### Target Information and Communication Task

After the attitude and entitativity manipulations were introduced, the target information was presented with a reference sheet. Several online consumer reviews were included in the reference sheet. Participants were told that the consumer reviews were collected from several online shopping sites. As mentioned above, this reference sheet on the target laptop consisted of four evaluatively ambiguous descriptions (including design, price, performance, and quality). After reading the reference sheet, participants proceeded to the communication task, which prompted them to type and send a message describing the information they had just read about the target laptop. The communication task was presented via an online survey using Google Forms. The reference sheet, evaluation sheet, and discussion memo were recovered before the communication task so that the participants did not refer to these materials during the communication task.

#### Follow-Up Measures

After the communication task, participants were asked to spend 10 min completing a number place puzzle (see Supplementary Figure 6). Similar to the tasks introduced in previous SIB studies, the number place puzzle allowed the decay of short-term memory for information about the target laptop. Participants then answered questions about the target laptop (recall and impressions) and the audience group (epistemic trust and entitativity) via another online survey using Google Forms. Participants were instructed to recall the original description of the laptop and report their impressions of it. For the recall task, participants were asked to reconstruct the original information described in the reference sheet in a free-recall format. For the impression measure, participants were then asked to type a few sentences that described their impressions of the target laptop.

After the recall and impression tasks, participants rated their epistemic trust in the audience group’s attitude toward the target laptop (see Echterhoff et al., 2005, 2008) through the item, “Is your addressee a person whose judgment about the laptop you can trust?” along with three other items (α = 0.77) which were rated using a 5-point scale (1 = not at all to 5 = very much). For entitativity measure, participants were asked how they perceive their audience group (Newheiser et al., 2012) through the item “The group is important to each member” and six other items (α = 0.74) which were rated using a 5-point response scale (1 = strongly disagree to 5 = strongly agree). In addition, to determine the success in manipulating the audience group’s attitude, participants were asked to recall whether their audience liked or disliked the target laptop from 1 (negative) to 10 (positive).

Finally, all participants were thanked and debriefed. In an after-experimental suspicion check during the debriefing, participants were asked whether they had any suspected rationale for the experiment, and then they were dismissed from the experiment.

#### Measures

To obtain the valence of the message, recall, and impression, two coders blind to the experimental conditions are required for coding, according to most SIB studies (e.g., Higgins and Rholes, 1978; Hausmann et al., 2008). Hence, we recruited two undergraduate students to undertake coding. Both coders were male due to the present status of application. Each protocol was broken into parts corresponding to the eWOM information in the reference sheet and assigned scores for positive or negative distortions on a 7-point scale ranging from −3 (extremely negative) to + 3 (extremely positive). As the audience attitude manipulation emphasized that the audience had been focused on design and price of the target laptop (e.g., good in design or too expensive) (see Supplementary Figures 2, 3), most participants ignored the other aspects (performance and quality) when they tried to share a message with the audience. Thus, the scores of design and price in the message protocols were used to rate the message valence. The overall scores were used to rate the valence of recall and impression protocols. The message valence and impression valence scores from the two coders were sufficiently correlated (*r*s = 0.81, 0.77); however, the intercoder correlation of recall valence was medium (*r* = 0.61). The means of the coders’ ratings were standardized, and the standardized values served as dependent variables in subsequent analyses.

## Results

### Manipulation Check

Participants in the positive audience attitude condition rated their audience’s attitude toward the target laptop as more positive (*M* = 6.72, *SD* = 1.28) than participants in the negative audience attitude condition (*M* = 5.27, *SD* = 1.51), *t*(49) = 3.70, *p* = 0.01. Thus, participants were aware of their audience’s attitude toward the target laptop, as expected.

We also confirmed that participants perceived the high entitativity group vs. the low entitativity group differently. As expected, participants in the high entitativity condition perceived higher entitativity of the audience group (*M* = 3.10, *SD* = 0.67) than did participants in the low entitativity condition (*M* = 2.38, *SD* = 0.62), *t*(49) = −4.02, *p* = 0.01.

### Audience Tuning

**FIGURE 2:**
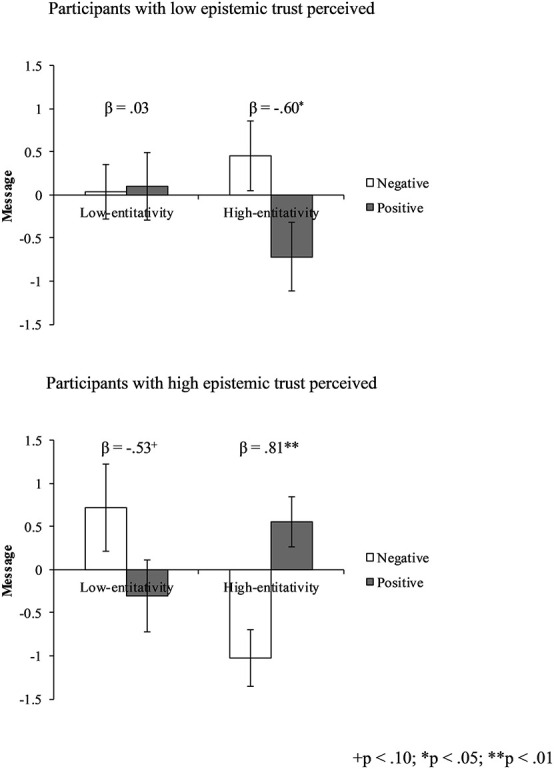
Effect of audience attitude, audience group entitativity, and epistemic trust on message.

To test hypothesis 1, we conducted a hierarchical multiple regression analysis with audience attitude, audience group entitativity, and epistemic trust as independent variables, and message valence as the dependent variable (see Table 1). In Step 1, no significant main effect of audience attitude, audience group entitativity, or epistemic trust was found. Results in Step 2 showed no significant two-way interactions. However, results in Step 3 revealed a significant three-way interaction between audience attitude, audience group entitativity, and epistemic trust. Following the procedure of Aiken and West (1991), we plotted the simple slope for each level of epistemic trust, and found that at higher levels of epistemic trust, participants created more positive (or negative) messages about the target laptop for the more entitative-positive (or negative) audience group. Participants who had low epistemic trust in their audience group did not tune their messages to the audience’s attitude (see Figure 2). Thus, Hypothesis 1 was supported.

**TABLE 1:** Moderated multiple regression analysis with message valence as a function of audience attitude, audience group entitativity, and epistemic trust (non-standardized regression coefficient).

**Measures**	**Step 1**	**Step 2**	**Step 3**
Audience attitude	−0.02	−0.02	−0.14
Audience group entitativity	−0.11	−0.08	−0.32
Epistemic trust	0.07	0.01	0.01
Audience attitude × audience group entitativity		0.61	0.69
Audience attitude × epistemic trust		0.64	0.62
Audience group entitativity × epistemic trust		0.17	−0.18
Audience attitude × audience group entitativity × epistemic trust			2.86**
*R* ^2^	0.00	0.09	0.29*
Δ*R*^2^	0.00	0.08	0.21
Δ*F*	0.07	1.27	12.04**

*p < 0.05; **p < 0.01.

### Recall and Impressions

To test hypothesis 2, we repeated the hierarchical multiple regression analysis to examine audience tuning as mentioned above, with recall valence and impression valence as dependent variables. For recall valence, results in each step revealed no significant main effect, two-way interaction, or three-way interaction (see Table 2).

**TABLE 2:** Moderated multiple regression analysis with recall valence as a function of audience attitude, audience group entitativity, and epistemic trust (non-standardized regression coefficient).

**Measures**	**Step 1**	**Step 2**	**Step 3**
Audience attitude	−0.09	−0.11	−0.11
Audience group entitativity	0.14	0.12	0.13
Epistemic trust	−0.32	−0.27	−0.27
Audience attitude × audience group entitativity		−0.08	−0.09
Audience attitude × epistemic trust		−0.46	−0.46
Audience group entitativity × epistemic trust		−0.62	−0.60
Audience attitude × audience group entitativity × epistemic trust			−0.13
*R* ^2^	0.05	0.12	0.12
Δ*R*^2^	0.05	0.07	0.00
Δ*F*	0.86	1.10	0.02

**FIGURE 3:**
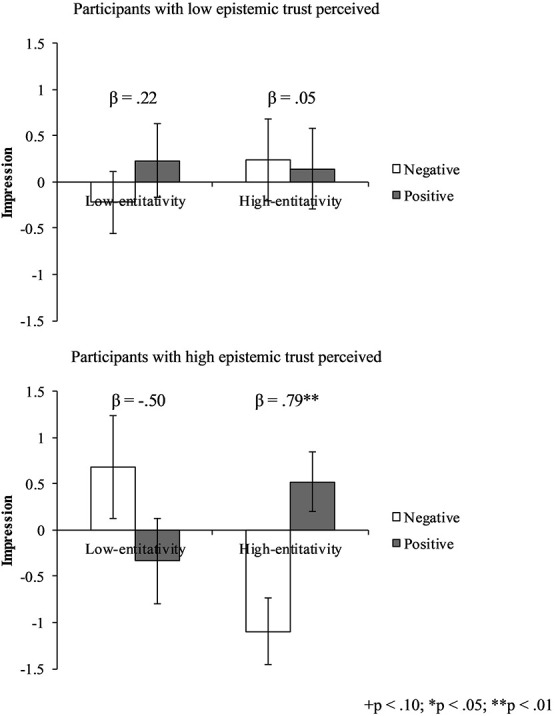
Effect of audience attitude, audience group entitativity, and epistemic trust on impression.

For impression valence, results in Steps 1 and 2 showed that neither the main effect nor the two-way interaction had a significant effect. However, in Step 3, a significant three-way interaction between audience attitude, audience group entitativity, and epistemic trust was found (see Table 3). The results for simple slopes showed that participants who communicated to a positive (vs. negative) audience group with high perceived entitativity evaluated the target laptop more positively (vs. negatively) when they had higher epistemic trust in their audience group, while participants who had low epistemic trust in their audience group did not (see Figure 3). Thus, Hypothesis 2 was partially supported.

**TABLE 3:** Moderated multiple regression analysis with impression valence as a function of audience attitude, audience group entitativity, and epistemic trust (non-standardized regression coefficient).

**Measures**	**Step 1**	**Step 2**	**Step 3**
Audience attitude	0.37	0.33	0.24
Audience group entitativity	0.07	0.08	−0.14
Epistemic trust	−0.13	−0.12	−0.11
Audience attitude × audience group entitativity		0.94	1.04^+^
Audience attitude × epistemic trust		0.12	0.09
Audience group entitativity × epistemic trust		−0.21	−0.48
Audience attitude × audience group entitativity × epistemic trust			2.37*
*R* ^2^	0.03	0.08	0.21
Δ*R*^2^	0.03	0.05	0.13
Δ*F*	0.52	0.83	6.92*

^+^*p* < 0.10; *p < 0.05.

## Discussion

People’s subsequent memory and impression of a communication topic can be biased by tailoring messages to their audience, which is the “saying-is-believing effect” (Higgins and Rholes, 1978). This study examined the SIB effect by focusing on a particular situation (e.g., eWOM communication) in which people often communicate with a large audience. In particular, we explored whether the perception of an audience as a group would moderate the SIB effect. As proposed, perception of an audience as a group may be affected by two factors: the degree to which a consensual viewpoint among the audience group can be identified, which is defined as entitativity, and the degree to which the viewpoint is considered trustworthy, which is defined as epistemic trust. Our results supported this proposition; the interaction between entitativity and epistemic trust significantly affected communicators’ message production and subsequent impressions of the topic. Awareness of a consensual viewpoint among the audience group encouraged audience tuning and led to bias in subsequent impressions only when the audience group was considered sufficiently trustworthy.

Note that while previous SIB studies have shown significant main effects of audience attitudes on audience tuning, recall or impressions, no similar main effects were found in the present study. This discrepancy may be attributed to the audiences explicitly included in this study that were perceived to have low entitativity and epistemic trust. In other words, in support of our proposition, we could infer that there was no main effect of audience attitudes on audience tuning or SIB for a large audience because of the moderated effect of perceived entitativity or epistemic trust.

This study was designed to explore whether the SIB effect would differ depending on how the communicator perceived an audience group. Extending prior SIB findings that focused on communication when the audience was a single person, Hausmann et al. (2008) demonstrated that the SIB effect would also occur when the audience comprised several persons under a particular condition, where communicators were provided with explicit feedback that all members of the audience group understood their messages. This finding indicates that the SIB effect is more likely to occur when the audience is perceived to have a consensual viewpoint. Based on the assumption that an overall group is more likely to hold similar viewpoints toward a specific topic, we proposed that the extent to which the audience is perceived as a group may affect the extent to which it is perceived to have a consensual viewpoint, which in turn affects the likelihood of the audience tuning and SIB effect occurring. Our results suggest that perceiving a higher degree of entitativity drives communicators to tune their messages to an audience group’s specific viewpoint, which leads to bias in subsequent impressions, but this is more likely when the audience group’s viewpoint is considered trustworthy.

Although our results revealed that the interaction between entitativity and epistemic trust moderates bias in subsequent impressions related to audience tuning, we did not obtain an expected effect for bias in subsequent memory. A possible reason for the unexpected result may be the insufficient intercoder correlation of recall valence. Another possible reason may be the modifications of the standard SIB paradigm, which involved descriptions of audience attitude manipulation and ambiguous descriptions for recall. In previous SIB studies, the audience attitude manipulation was introduced by simply mentioning that the audience either liked or disliked the target person, and the ambiguous recall descriptions included four detailed descriptions of the target person. It is possible that the audience attitude manipulation descriptions were not directly associated with ambiguous descriptions of recall in the standard SIB paradigm. However, there may be a direct association between the audience attitude manipulation descriptions and the ambiguous descriptions for recall related to the paradigm modifications. With the audience attitude manipulation, we presented concise information through an evaluation sheet comprising two simple descriptions (information related to price and design), which referred to the audience group that either positively or negatively evaluated the target laptop. In response to the ambiguous recall descriptions, we presented a reference sheet with four detailed descriptions (information related to performance, price, design, and quality) about the target laptop. The direct association between audience attitude manipulation descriptions and recall (related to price and design) may have triggered higher accessibility for those specific descriptions; it may also have suppressed participants’ use of indirectly associated descriptions (related to performance and quality). Since Martin et al. (2001) demonstrated that the judged availability of activated information is an important precondition for the use of this information, communicators in our experiment may have judged indirectly associated descriptions as irrelevant or inappropriate to their tasks. That is, communicators may have neglected information about performance and recall quality. When the recalled descriptions were broken into parts by the coders to rate the overall valence, no scores could be assigned to neglected information. Therefore, we assumed that the assigned scores representing the recall distortions might be incomplete, and this might be an important factor behind the unexpected recall valences.

The current research demonstrates how the perception of an audience group affects interpersonal communication. By extending previous research, we advanced knowledge on how communication affects communicators’ attitudes. In addition, we addressed the possibility of new discussions in the eWOM literature by employing psychological concepts and paradigms.

Previous research has demonstrated that the SIB effect occurs when people experience a shared reality with their audience. These studies manipulated the extent of the experienced shared reality by informing communicators with (vs. without) explicit feedback that the audience understood their messages (Hausmann et al., 2008), or by applying explicit success (vs. failure) feedback (Echterhoff et al., 2005). However, we argued that a discussion of phenomena involving the SIB effect should include not only the experienced shared reality, but also the expectations of successful shared reality. From the perspective of successful shared reality expectations, we demonstrated that the SIB effect occurs depending on the communicators’ perception of entitativity and epistemic trust in a large audience. In our study, the extent of successful shared reality expectations was only conceptually explained, and not directly measured or manipulated. Therefore, it is necessary to manipulate the expectations of successful shared reality directly and to verify the validity of this explanation in future studies.

Future research should also investigate whether intergroup relations between communicators and audiences influence the SIB effect by examining whether a difference between communicating with an in-group and an out-group affects communicators’ audience tuning. Bias in subsequent memory and impression should also be discussed, along with entitativity. The findings of our research, which was focused on group perception, reflect the unique psychology underlying communication with a large audience and is consistent with Hamilton and Sherman (1996) research on impression formation. However, we recognize that group perception may also be based on group membership—in-group and out-group recognitions. Previous research has shown that being aware of group membership may also affect epistemic trust in the audience (Hewstone et al., 2002). Furthermore, a recent SIB study that examined how intergroup communication plays a role in producing the SIB effect also suggests that communicating with an in-group vs. an out-group can drive the difference in epistemic input (e.g., epistemic trust), which in turn can lead to differences in SIB effect output (Echterhoff et al., 2017). Therefore, it would be interesting to explore whether entitativity perception moderates the SIB effect in intergroup communication. Varying the entitativity of the audience group (as a mere collection of individuals or a unified group) and intergroup (as in-group or out-group) might yield useful information on the SIB effect. Therefore, future studies should examine this issue in greater detail.

## Data Availability Statement

The original contributions presented in the study are included in the article/Supplementary Material, further inquiries can be directed to the corresponding author/s.

## Ethics Statement

The studies involving human participants were reviewed and approved by Ethics Committee of Hiroshima University, Graduate School of Humanities and Social Sciences. The patients/participants provided their written informed consent to participate in this study.

## Author Contributions

All authors were involved in revising the manuscript, provided their approval for the publication of the manuscript, and agreed to be accountable for all aspects of the work, to ensure that questions related to the accuracy and integrity of any part of the work are appropriately investigated and resolved.

## Conflict of Interest

The authors declare that the research was conducted in the absence of any commercial or financial relationships that could be construed as a potential conflict of interest.

## Publisher’s Note

All claims expressed in this article are solely those of the authors and do not necessarily represent those of their affiliated organizations, or those of the publisher, the editors and the reviewers. Any product that may be evaluated in this article, or claim that may be made by its manufacturer, is not guaranteed or endorsed by the publisher.
