# Supplementary material for: How Group Perception Affects What People Share and How People Feel: The Role of Entitativity and Epistemic Trust in the “Saying-Is-Believing” Effect
Source: Front Psychol. 2021 Sep 22;12:728864. doi: 10.3389/fpsyg.2021.728864 (PMC8494462; doi:10.3389/fpsyg.2021.728864)
Supplement: Supplementary file 1 [file Table_1.pdf]

Supplementary Material

1 Supplementary Figures

【資料（３）：クチコミ情報】

Handouts (3): word-of-mouth

【クチコミ情報（ノートパソコン③）】（一部抽出）

[Word-of-mouth (about laptop No.3)] (partially extracted)

さん | クチコミ投稿数: 126件 Goodアンサー獲得: 2件 2017/09/07 16:02(2ヶ月以上前)

薄型軽量が特徴なので、外で使いたい人には便利だと思います。

キーボードが若干小さく打ちにくい感じがするので、文章作成などには不向きかもしれませんが。

It is thin and lightweight, so I think it is convenient for people who want to use it outside.
The keyboard is a little small and it feels difficult to type, so it may not be suitable for writing sentences.

さん クチコミ投稿数: 27228件 Goodアンサー獲得: 7158件 2017/11/08 01:03

値段はやはり（少し）高いと思うけど、ほかの軽量ノートに比べると比較的に安いです。

https://www.microsoft.com/ja-jp/surface/devices/surface-laptop/tech-specs

I think the price is (a little) expensive, but it's relatively cheap compared to other lightweight notebooks.

★★★☆☆ **購入しました。**
投稿者 2017年1月11日
サイズ: Core i7 | 色: 1TB | で購入

本体色のセンスは少しダサい感じがします…デザイン自体がシンプルでかなり洗練されていますが…

The sense of body color feels a little dull ... Although the design itself is simple and quite sophisticated ...

★★★☆☆ **とにかく小さくて安くて軽いのがよければ**
投稿者 2017年4月5日
で購入

大手メーカーの製品だけで高品質なものだろうけど、
薄型になっているから落下などの衝撃に弱い面もあると思います。なので☆３つで。

It's probably high quality only from the products of major manufacturers,
Since it is thin, I think it is vulnerable to impacts such as dropping. So I rated with 3 stars.

Supplementary Figure 1. Four evaluatively ambiguous descriptions of the target laptop. [Note that the original descriptions in appendix 1 as well as in other appendixes were presented in Japanese in our experiment. English translations (in the text boxes) were added for reference.]

Audience attitude: positive

【資料（１）：評価メモ】

Handouts (1): evaluation sheet

ノートパソコン③

Laptop No.3

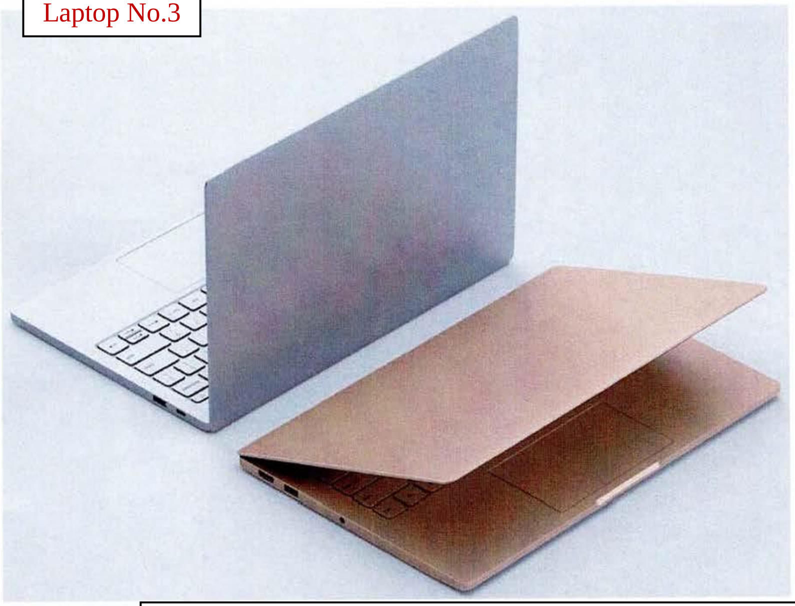

(Please specify the evaluation you most sympathize with O.)

MEMO（最も共感している評価を○などで明記してください。）

Performance	性能	普通	Average
Price	価格	よくわからない。	Not sure
Design	デザイン	良い ○ ○ ○ ○ ○ ○	Good
Quality	品質	よくわからない。	Not sure
Others	その他		

Supplementary Figure 2. Evaluation sheet used for audience group attitude manipulation, which indicated positive audience attitude.

Audience attitude: negative

【資料（１）：評価メモ】

Handouts (1): evaluation sheet

ノートパソコン③

Laptop No.3

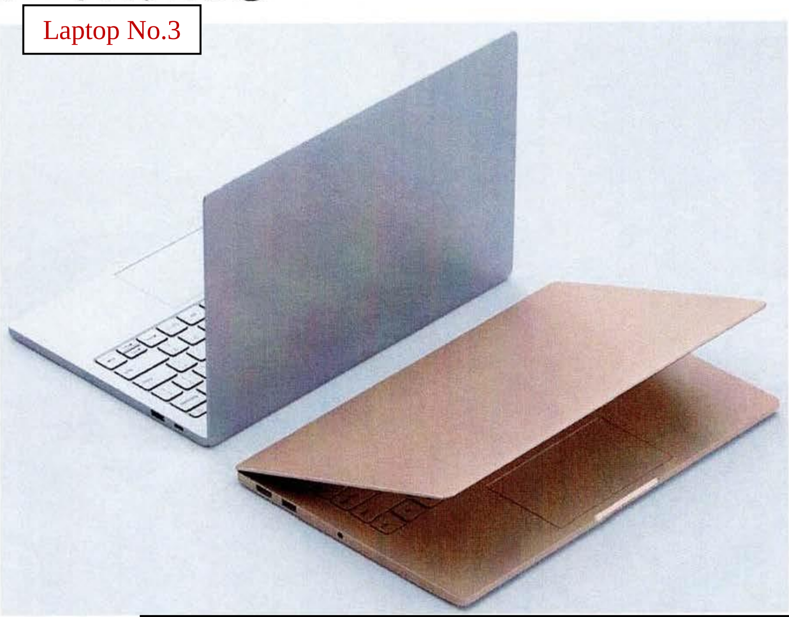

(Please specify the evaluation you most sympathize with O.)

MEMO（最も共感している評価を○などで明記してください。）

Performance	性能	普通	Average
Price	価格	高すぎ ○ ○ ○ ○ ○ ○	Too expensive
Design	デザイン	よくわからない	Not sure
Quality	品質	よくわからない.	Not sure
Others	その他		

Supplementary Figure 3. Evaluation sheet used for audience group attitude manipulation, which indicated negative audience attitude.

Audience group entitativity: high

【資料（２）：議論の様子】

Handouts (2): discussion memo

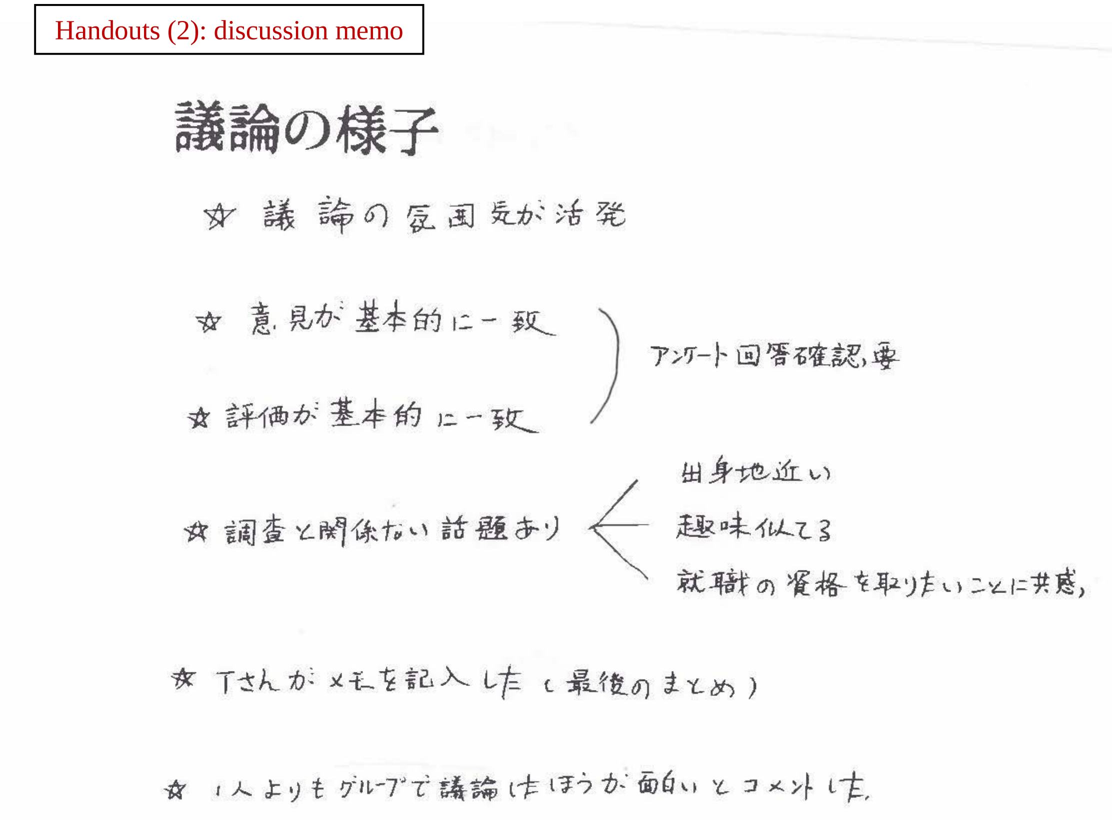
議論の様子

☆ 議論の雰囲気が活発

☆ 意見が基本的に一致

☆ 評価が基本的に一致

アンケート回答確認、要

☆ 調査と関係ない話題あり ←

出身地近い

趣味似てる

就職の資格を取りたいことに共感,

☆ Tさんがメモを記入した（最後のまとめ）

☆ 1人よりもグループで議論したほうが面白いとコメントした.

Discussion memo

The atmosphere of discussion is lively.

Their opinions are basically in agreement.

Need to confirm the answers to the questionnaire

Their evaluation ratings are basically in agreement.

There were conversation topics unrelated to this survey.

Their hometowns were near.
Their hobbies were similar.
They have the same goal for getting a job qualification.

T (person's name) wrote this memo (final summary).

They commented that it was more interesting to discuss in this group than to think alone.

Supplementary Figure 4. Discussion memo used for audience group entitativity manipulation, which indicated high entitativity group properties.

Audience group entitativity: low

【資料（２）：議論の様子】

Handouts (2): discussion memo

議論の様子

☆議論の雰囲気があまり盛り上がらない。

☆意見がバラバラ

☆評価がバラバラ

→ アンケート回答確認要

☆調査と関係ない話題あり
- 出身地が違う
- 趣味バラバラ
- 就職・進学など目標が違う

☆Tさんがメモを記入した（最後のまとめ）

☆グループよりも1人で評価したほうがやりやすいとコメントした。

Discussion memo

The atmosphere of discussion is not lively.

Their opinions are different.

Their evaluation ratings are different.

→ Need to confirm the answers to the questionnaire

There were conversation topics unrelated to this survey.
- Their hometowns are far away.
- Their hobbies are different.
- They have different goals for getting a job or going on to higher education

T (person's name) wrote this memo (final summary).

They commented that it would be easier to think alone than to discuss in this group.

Supplementary Figure 5. Discussion memo used for audience group entitativity manipulation, which indicated low entitativity group properties.

Student number

Grade

Sudoku test (10 minutes)

学生番号 ______________　　学年 ________

Gender　性別　男・女　　年齢 ________

Male/Female　　Age

この数独テストは大学生を対象に行い、集中力・思考力と学年との関係を検討することを目的とします。

This Sudoku test aims to examine the relationship between concentration/thinking ability and grade for colleges students.

【ルール】(1)　空いているマスに 1〜9 のいずれかの数字を入れる。
(2)　縦・横の各列及び、太線で囲まれた 3×3 のブロック内に同じ数字が複数入ってはいけない。

Rules: (1) Enter any number from 1 to 9 in the empty space.
(2) The same number must not be entered more than once in each of the vertical and horizontal columns and in the 3x3 block surrounded by thick lines.

5	3			7				
6			1	9	5			
	9	8					6	
8				6				3
4			8		3			1
7				2				6
	6					2	8	
			4	1	9			5
							7	9

※数独テストを完成できたら、担当者の指示があるまで待っていてください。

* When you have completed the Sudoku test, please wait until you receive the next instruction.

Supplementary Figure 6. Number place puzzle which allowed the decay of short-term memory for information about the target laptop.
